# Advances in human organoids-on-chips in biomedical research

**DOI:** 10.1093/lifemedi/lnad007

**Published:** 2023-02-21

**Authors:** Yaqing Wang, Jianhua Qin

**Affiliations:** Division of Biotechnology, Dalian Institute of Chemical Physics, Chinese Academy of Sciences, Dalian 116023, China; Division of Biotechnology, Dalian Institute of Chemical Physics, Chinese Academy of Sciences, Dalian 116023, China; Beijing Institute for Stem Cell and Regenerative Medicine, Beijing 100101, China; Suzhou Institute for Advanced Research, University of Science and Technology of China, Suzhou 215123, China; University of Chinese Academy of Sciences, Beijing 100049, China; CAS Center for Excellence in Brain Science and Intelligence Technology, Chinese Academy of Sciences, Shanghai 200031, China

**Keywords:** organoid, organs-on-chips, organoids-on-chips, stem cell, biomedical research

## Abstract

Organoids-on-chips is opening up new frontier of research in biomedical field by combining organoids and organs-on-chips technology. The integrative technology offers great opportunities to maximize the potentials of organoids with higher fidelity, thus building advanced organ model systems in a physiologically relevant manner. In this review, we highlight the key features of organoids-on-chips and how this integrative technology could be used to build organoids in higher fidelity under controlled cellular microenvironment. We then introduce the recent progress of organoids-on-chips and their applications in biomedical research. We also discuss the opportunities and challenges of the nascent field of organoids-on-chips that lie ahead to accelerate their utility in disease research, drug testing, and regenerative medicine.

## Introduction

Rapid progress in life science calls for new *in vitro* organ models to meet the urgent needs of current biomedical research and pharmaceutical applications. The development of next-generation of experimental models is of great significance for deep-understanding of human health and disease processes, and for designing effective therapeutics. Organoids are *in vitro* three-dimensional (3D) multicellular tissue constructs via self-organization of stem cells or tissue progenitors [1–4]. They can mimic human physiology more accurately and capture complexity of biological process compared with traditional 2D cultures and animal models, which have shown obvious advantages for their utility in organ development, disease modeling, drug discovery, and regenerative medicine [1, 5–10]. Despite the great potentials of organoids, existing organoid culture systems possess some limitations such as high variability, less maturity, and low controllability. Researchers are now starting to take inspirations from bioengineering and materials fields to generate organoids that are more physiologically relevant and amenable to targeted applications.

**Figure 1.**
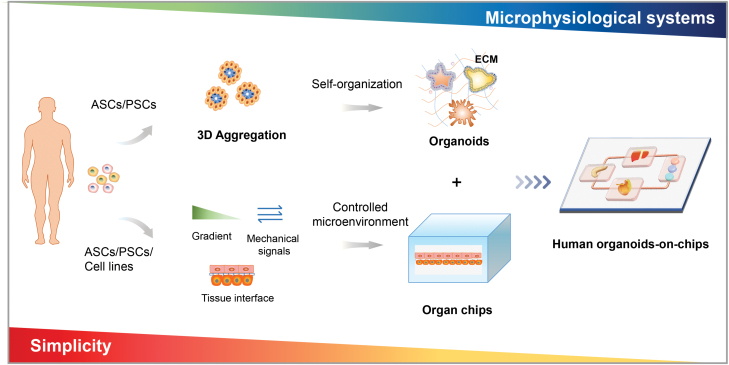
Schematic of engineered organoids-on-chips by combining organs-on-chips (organ chips) and organoids. Organoids are 3D multicellular tissues that derived from human PSCs (ESCs and iPSCs) or ASCs by self-organization. Organ chips can be utilized for engineering organoids by guiding stem cells differentiation, organization, and organoids formation in a controlled microenvironment, thereby improving functions and maturity of organoids for advancing biomedical research.

Considerable advances in microfabrication and microfluidic organ-on-a-chip (organ chips) technologies provide unprecedented opportunities to enable precise control of cell shape, position, function, and tissue organization in a highly arranged 2D or 3D structures. Organ-on-a-chip is a miniature cell culture device that contains perfused micro-channels inhabited by living cells [11–13]. It can recapitulate key aspects of architectural and functional hallmarks of tissues/organs by control over fluid flow, biophysical and chemical factors, and cell–cell/cell–matrix interactions. The remarkable progresses have been made to reconstruct various functional units of living tissues/organs-on-chip, such as the liver [14], kidney [15, 16], intestine [17], etc. Recently, the convergence of organ chips technology with organoids has led to the development of “organoids-on-chips” to produce organotypic models with more physiological relevance (Fig. 1) [18–21]. As a new frontier technology, organoids-on-chips can direct stem cell differentiation and organoid organization in a controlled cellular microenvironment. This technology could also integrate multiple analysis for accurately monitoring culture environment and organoid behaviors at multi-scales.

In this review, we summarize how the integrative organoids-on-chips strategy can be adopted to engineer organoids with better production, control, and functions. Then we introduce the recent progress on the development of organoids-on-chips. We also discuss the challenges and perspectives of organoids-on-chips to advance their applications in biomedical research.

## Snapshot of organoids

Organoid originates from the self-organization of stem cells or tissue progenitors in 3D matrix, the stem cells include embryonic stem cells (ESCs), induced pluripotent stem cells (iPSCs), and tissue-specific adult stem cells (ASCs). Generally, the formation of embryonic bodies (EBs) is an initial and critical stage for organoid production in 3D culture. EBs can be further differentiated into defined germ layer, followed by specific organoids that resemble their tissue of origin. This process recapitulates the key events of organogenesis during early development, including self-renewal and spatial cell-lineage differentiation [22]. A pioneering work of intestinal organoid derived from human ASCs perhaps opened up the organoid field in 2009 [23], which demonstrated the intrinsic capacity of stem cells to self-organize and mimic the native organ. Recently, multiple types of stem cell organoids have been successfully generated to delineate the physiological hallmarks of human developing organs, such as the brain [24, 25], intestine [23], retina [26], lung [27], liver [28–30], pancreas [31], and kidney [32]. In addition, organoids derived from patient stem cells or generated by introducing disease mutations can be used to model genetic disorders and study disease pathogenesis, extending their translational applications in drug testing and personalized medicine.

The derivation of organoids mainly relies on several approaches in 3D cultures, including EBs or cell aggregates embedded in extracellular matrix (ECM) [24, 27, 33–36], suspension cultures [6, 23, 25, 37], and the air–liquid interface cultures [38, 39]. Generally, most organoids rely on the self-organization of stem cells within animal-derived 3D matrix (often Matrigel) in a dish. It is noteworthy that existing organoid models often represent only single or partial properties of native tissues, and still remain some limitations. One is the high variability of architecture, phenotypes, and cellular composition in organoids, which might be partially due to the lack of defined 3D matrix. For example, the batch-to-batch variations and ill-defined protein compositions of Matrigel may lead to high variability in organoids. Moreover, the random process of organoid self-organization can lead to the uncontrolled microenvironment. Second, most organoids lack multicellular (e.g., immune cells, innervation, and blood vessel structure) or organoid–organoid interactions that are critical for morphogenesis and functional maturity of organoids. Third, the organoid culture systems often require tedious manual operation and show low analytical flux, which may hinder their translational applications.

## Integrative strategies to engineer organoids-on-chips

**Figure 2.**
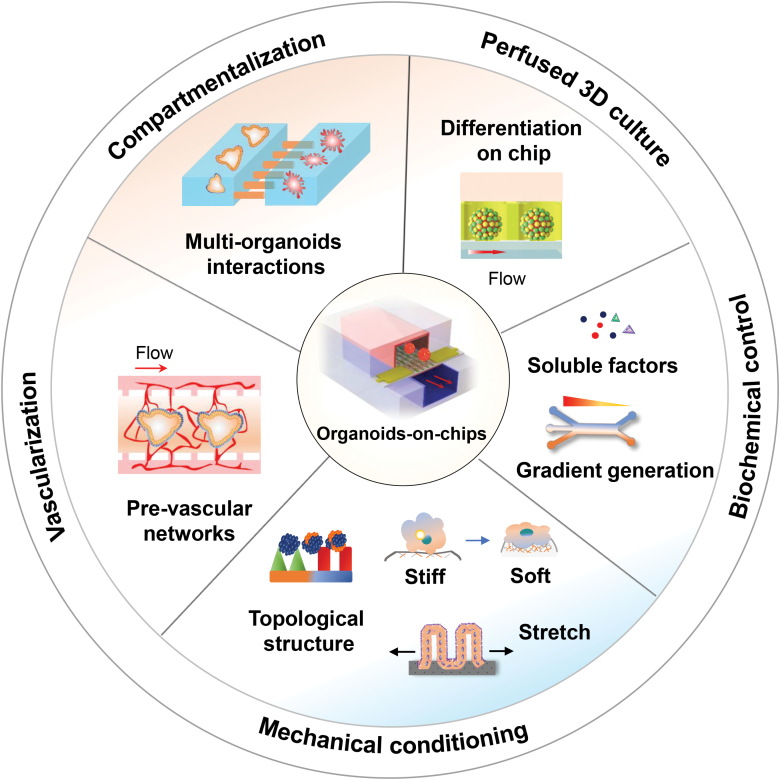
Illustration of integrative strategies to engineer advanced organoids-on-chips. The perfused 3D culture on chip may facilitate nutrient exchange and long-term survival of organoids. The spatiotemporal control of the biochemical components including growth factors gradients may guide organoid formation resembled *in vivo*. The spatiotemporal control over the biophysical microenvironment cues, such as stretch, topological structure, and substrate stiffness may steer organoid differentiation and formation during development. The incorporation of pre-vascular networks on chip may facilitate the vascularization of organoids. Organ chips with compartmentalized microenvironments may allow co-culture of different types of organoids, recapitulating the complex interactions in organism.

As above, the limitations of existing organoid systems have provided an impetus for the development of new strategies that better mimic the complex structures and functions of living organs. Organoids-on-chips originate from the rapid development of organ chips technology. It can mimic near-physiological tissue microenvironment by integrating the advantages of organoids and organ chips technology, thereby instructing stem cell behaviors and organoid morphogenesis. Organoids-on-chips may provide a new path to engineer organoids in a physiologically relevant manner. In the following section, we will discuss how the integrative strategy can be utilized to advance organoid model systems (Fig. 2).

### Perfused 3D culture

3D architecture of an organoid plays a crucial role in modeling *in vivo* organogenesis [36]. The establishment of SFEBq (serum-free culture of EBs with quick re-aggregation) in suspension 3D culture approach or embedded in Matrigel droplet has enabled the formation of organoids in 3D cultures [37, 40]. In addition, the agitation cultures have been used for long-term culture of organoids in a spinning bioreactors [24, 25], however, the uncontrolled flow or shear stress may lead to abnormal differentiation and limited maturation. The implementation of microfluidic perfusion networks could be a potential strategy to address this issue. Organoids-on-chips could enable perfused 3D culture of organoids by the design of defined culture chambers and precise control of continuous flow. Recent studies have demonstrated the significant role of fluid flow in the development of organoids. For example, the administration of continuous media flow facilitated the formation of polarized intestinal organoids with villi-like structures that contained multiple epithelial subtypes [41, 42]. Tao et al. found the perfused cultures enable enhanced cell viability in islet organoids derived from human iPSCs (hiPSCs) on chip and more sensitive insulin-secretion functions by regulating calcium flux [43]. The similar strategy was also used to generate liver and brain organoids derived from hiPSCs, which facilitated their long-term culture and functional maturity [44, 45]. In short, these studies highlight the great potential of organoids-on-chips technology to create perfused 3D culture system, which contribute to controllable production and development of organoids.

### Controlled tissue microenvironment

*In vivo*, the development of tissues or organs during embryogenesis is dynamically regulated by the spatiotemporal control of inherent genetic reprogram of cells and the local niche cues. Generally, stem cells were exposed to extrinsic morphogens or growth factors at defined time points to induce differentiation and organoid formation. These signals can trigger organoid organogenesis with the activation of desired developmental signaling pathway. It is noted that organoid morphogenesis is also tightly controlled by the external microenvironment cues, including biochemical factors (cytokines, morphogen gradients) and physical factors (mechanical forces, electrical signals) [4, 46]. As such, towards advanced 3D tissues and organs, it is essential to improve organoids generation by precisely spatiotemporal control of physiological microenvironment cues.

#### Morphogen gradients formation

*In vivo*, body axis is established through the concerted action of morphogen gradients, such as the ventral–dorsal axis that allows the organization of heterogeneous brain tissues with diverse regions along the axis. Therefore, to generate temporally and spatially defined soluble factor gradients are essential in directing differentiation and formation of heterogeneous organoids. The general method of microfluidic gradient generation is chamber systems by diffusion-based source and sink of chemical input [47, 48]. For example, a microfluidic system was reported to generate physiological morphogen gradients to model neural tube development [49]. The designed microfluidic chip can generate opposing and/or orthogonal gradients of morphogens for ESCs culture and differentiation into neural tube analog under continuous fluid flow. Recently, Rifes et al. generated a neural tissue exhibiting hallmarks of rostro-caudal organization using a WNT-activating gradient in a microfluidic device, modeling early human neural tube development [50]. The microfluidic-based gradient generator provided a new platform to guide stem cell differentiation and organoid organization along the axis by spatiotemporal control over the chemical environment.

#### Mechanical conditioning

The mechanical stimuli, such as rigidity, tensile deformation, and topography, have broadened many horizons about in-depth comprehension of cell behaviors, including adhesion, motility, deforming, differentiation, and assembling into desired tissues. Organoids-on-chips could simulate the mechanically active microenvironment of tissues by administration of various biological forces. For example, the luminal flow through human gastric organoids allowed to rhythmically introduce stretch and contraction to the organoid-on-chip, mimicking *in vivo* gastric motility [51]. In addition, electrophysiological stimulation has been interwined with cardiac tissue in order to enhance maturity in modern cardiac research. A new platform-biowire on PDMS channel with electrical stimulations has been developed to markedly increase cardiomyocyte organization and electrophysiological properties [52]. Moreover, electrical conditioning was recently reported to facilitate the formation of distinct atrial and ventricular tissues [53]. Organoids-on-chips can also provide scaffolds with specific substrate topology or internal structure via photolithography, as well as chemical and physical pattern. The topological structure could offer specific microenvironment to guide the formation of organoids with defined architecture that resembling targeted tissue and reducing variability. For example, researchers fabricated 3D scaffolds matching the architecture of intestinal crypt and villus to reproduce intestinal epithelium topography, which may facilitate guided self-organization and controlled differentiation of organoids [54, 55]. Furthermore, chips with appropriate geometric configurations, such as micropillar and microwell array have been fabricated to control the formation of EBs with uniform size and *in situ* differentiation of organoids [19, 44, 56], thereby reducing the variability of organoids and greatly simplified the tedious organoid-derivation procedures. It also provides a low-cost and flexible platform to address a current limitation of large-scale production of organoids to some extent. In addition, the confined internal structure offered by chip channels holds promise to discover the fundamental mechanisms of organoid morphogenesis associated with mechanical forces. For example, engineered brain organoid in confined compartment of a microfabricated chip was used to model the biomechanics of cerebral folding, revealing the biological and physical mechanism of surface wrinkling during early brain development [57].

As biomimetic matrix, hydrogels served as substrate are often integrated with microfluidic system for constructing 3D tissues. Shaping surface topography or regulating hydrogel stiffness may also be used to guide cell alignment and differentiation into organoids with specific architecture resembled *in vivo*. For instance, a perfused chip platform contained collagen-based hydrogels with suitable stiffness was fabricated to shape intestinal organoids. It was used to create a polarized intestinal epithelium crypt-villus architecture with appropriate cell-lineage compartmentalization under luminal perfusion, recapitulating the key features of native small intestine [58]. This microengineered approach provided a physiologically relevant microenvironment to control the self-organization of stem cells into intestinal organoids *in vitro*, which may also be applicable to construct other organoid models with more complex structure and physiological function.

### Vascularized organoids

Vascularization of organoid is tightly associated with organoids maturation *in vitro*, which is a critical issue to be solved in organoids research. *In vivo*, organs consist of hierarchically vascular networks that ensure organ development and maturation by delivering adequate nutrients and oxygen [59, 60]. Based on this, organoids-on-chips integrated with vascular networks is necessary to address the issue of nutrient diffusion limitation and less maturity in current organoid systems. Fluidic shear stress (FSS) may be a key environmental factor for the construction of vascular architecture by regulating endothelial cell functions [61, 62]. For example, Homan et al. generated vascularized kidney organoids in a closed-loop millifluidic chip under FSS [63]. They demonstrated that high FSS (0.008–0.035 dyn/cm^2^) enhanced abundance of vasculature and form perfused vascular system via the activation of endogenous angiogenic pathways, which increased tissue functionality and maturity of kidney organoids. This method may be applicable to other organoid types during development under controlled fluid flow, which might be beneficial to generate more functional organ equivalents. In addition, the integration of endothelial or progenitor cells within organoid systems may also induce new blood vessels formation and assembling within organoid, such as the layer-by-layer manner [64, 65]. Chip device consisted of multichannels can allow uniform distribution of flow and mass transfer fabricated using soft lithography. Bioprinting methods can then be used to seed various vascular cells in the channels, followed by creating perfusable vascular structures for organoids culture. In these approaches, ECM scaffold is a key parameter to collectively determine the formation of vascular structure combined with fluid flow. Chemically defined matrices have been used to create *in vitro* microengineered 3D vessel networks in perfusable microfluidic chip due to their tunable biochemical and mechanical properties [66, 67]. It is noted that these strategies will need to be modified in organoid systems that allow specialized physiological functions.

### Tissue–tissue interactions

*In vivo*, individual organs almost never develop in isolation, but rather concurrently with surrounding organs. The interactions among organs are essential for organogenesis. To combine multiple organoids in a single system is vital to recapitulate the systemic inter-tissue or inter-organ communications, which is crucial for organ development, as well as accurate drug and therapeutic studies. Organ chips technology has shown promise to establish the functional connection of different tissues with flexible design [68, 69]. For example, Trapecar et al. established a multi-organ chip integrated with gut, liver, and brain tissues to study systemic interactions in the context of neurodegenerative diseases [69]. The dynamic flow has been demonstrated to support the interactions among diverse tissues via the transport of excretive soluble factors resembling *in vivo* vasculature. This would facilitate the reconstruction of organ–organ boundaries, thus elucidating the complex physiology and pathology of human organism. Recently, a multi-organ chip with matured human heart, liver, bone, and skin tissue niches linked by vascular flow was developed [70]. It recapitulated interdependent organ functions in the presence of endothelial barriers and used for drug testing.

In addition, the interactions of multiple cell lineages are critical for organoid dynamics and proper morphogenesis. The lack of important physiological interactions, such as vascularization and innervation, may limit the complexity and maturation of organoids. Organ chips make it possible to reconstruct these elements and interconnections by co-culture with different cell types or tissues, recapitulating the cellular interactions during organogenesis. Besides the tissue–tissue or multicellular interactions, host–microbiota interaction is also a critical factor for the regulation of tissue-specific homeostasis and controlled microenvironment, e.g., the interplay between the intestine and microbiota [71, 72]. Moreover, co-cultured with immune cells/pathogens and organoids-on-chip could more precisely reflect the host–immune or host–pathogen interactions, which may contribute to construct robust drug predictive models and application of personalized medicine.

### High-throughput analysis

To achieve the full utility of organoids, it is also necessary to analyze the multi-parameter signals and multi-dimensional information from organoids models, which is lacking in current organoid systems. The functional readout system will help to automatically control organoid culture and better understand the effects of microenvironment parameters on organoids development. The integration of multiplexed biosensors, such as oxygen sensors and electrical arrays, is potential solutions [73, 74]. For example, Zhang et al. reported a multiple organoid models integrated modular physical, biochemical, and optical sensing sensors into modularized organ chip unit for automatic and *in-situ* continual monitoring organoid behaviors and biochemical parameters [75]. This platform provides a new platform for the study of interactions between multiple tissue/organs and drug testing. In addition, Tay et al. [76] developed an automated microfluidic platform for human-derived tumor organoids culture and real-time analysis of their responses to drug treatments. The integrated system hold promise to advance organoids models for high-throughput drug screening and personalized therapy.

## Recent progress of organoids-on-chips

Recently, organoids-on-chips have made important progress and produced a variety of engineered organoids that are summarized in Table 1. These organoids-on-chips models promote the development and maturity of organoid with more complicated architectures and functions in controllable stem cell microenvironment. Moreover, they have been initially used in applications of organ development, drug testing, and disease modeling, showing significant prospects in the field of life medicine. In this section, we outlined recent progress of organoids-on-chips that would deliver on the promise of research in human physiology and pathology.

**Table 1.** Summary of recent progress of human organoids-on-chips and their applications

Types of organoids-on-chips	Cell sources	ECM	Chip design	Key hallmarks of organoids	Applications
Brain	hiPSCs [19, 20, 45, 56, 77, 78]	Matrigel	A high throughput micropillar array chip [19, 56] A perfusable chip system contained parallel multichannels [20, 45] Microfluidic fabricated hollow alginate fiber [77, 78]	Controlled EBs formation High-throughput generation Perfused 3D culture in matrix *In situ* neural differentiation and brain organoids formation on a single device Recapitulating neuronal differentiation, brain regionalization, and cortical organization	Modelling brain development and exposure of prenatal environmental factors (e.g., nicotine, alcohol, and cadmium) exposure at the early stage
	hESCs [57]	Matrigel	A microfabricated compartment contained a top polycarbonate membrane and a bottom coverslip	*In situ* imaging over a timescale of weeks	Revealing the physics of brain folding
Liver	hiPSCs [44, 79, 80]	None	A perfusable micropillar chip system [44, 80] A perfusable chip system with C-trap architecture [79]	Controlled EBs formation *In situ* differentiation and formation of organoids on a single chip Enhanced hepatic-specific functions in liver organoids	Drug testing Modeling human NAFLD
Intestine	hiPSCs [42]	Matrigel	A multilayer chip with a porous membrane Culturing intestinal organoid-derived epithelial cells under flow	Generating polarized intestinal folds that contained multiple epithelial subtypes	Modelling biologically responsive to exogenous stimuli
	hASCs [41, 58, 64]	Collagen I and Matrigel [58]	A multilayer chip with a porous membrane and cyclic deformation [41] A perfusable microchip system contained crypt-like microcavities and hydrogel scaffold [58] A customized 384-well plate-based IFlowPlate by an “open-well” design [64, 81]	Forming villi-like polarized structures that undergo multi-lineage differentiation Forming perfusable mini-gut tubes with a similar spatial arrangement of crypt- and villus-like domains to that *in vivo* Generating vascularized colon organoids	Generating intestinal closely mimics whole human duodenum *in vivo* Forming a polarized and dynamic intestinal organoid Modeling colon inflammation by inflammatory cytokine stimulation
Islet	hiPSCs [43, 82]	3D alginate hydrogel [82]	A perfusable multilayer chip containing a top microwell array, a polycarbonate porous membrane and a bottom PDMS layer [43] A microphysiological system (MPS) that enables continuous dynamic culture and *in situ* multiparametric assessment [82]	Controlled EBs formation, *in situ* pancreatic differentiation and islet organoids formation Exhibiting more sensitive glucose-stimulated insulin secretion and Ca^2+^ flux under flow Preservation of organoid function under dynamic microenvironment in MPS	Recapitulating the key cellular composition and functions of human islet organoids
Stomach	hPSCs [51]	Matrigel	A central chamber for culturing organoid and two in-line chambers for media	Allowing long-term and 3D culture of gastric organoids under luminal flow	Modeling gastric peristaltic motility as like *in vivo*
Retina	hiPSCs [83, 84] ESCs [85]	Matrigel	A chip containing top four tissue compartments connected via a microchannel and bottom perfused channel separated by a thin porous membrane [83, 84] A shear stress-free micro-millifluidic bioreactor containing linear single-sided chambers and serpentine alternating side chambers [85]	Recapitulating the interaction of mature photoreceptor segments with RPE by co-culture of retinal organoids and RPE Enabling enhanced inner and outer segment structures formation Modeling key functionalities of the visual cycle, such as calcium dynamics Enabling long-term culture of retinal organoids	Evaluating drug-induced retinopathy [84] Validating Adeno-associated virus retinal gene therapy vectors [83]
Kidney	hPSCs [63]	Gelatin and fibrin	A perfusable millifluidic chip by 3D bioprinting	Generating vascularized kidney organoids that exhibit more mature podocyte and tubular compartments under flow	Recapitulating the process of glomerular vascular development and morphogenesis of kidney organoids
	hASCs [86]	Collagen I	A three-lane OrganoPlate platform with parallel chips	Building polarized kidney tubules under flow Displaying trans-epithelial transporter activity	Modeling BK virus infection, malignant, and hereditary kidney diseases in a personalized fashion
Liver and islet	hiPSCs [87]	None	A perfused microfluidic multi-organoid system containing two compartmentalized regions connected by a microchannel network	Exhibiting favorable growth and improved tissue-specific functions of co-cultured organoids Recapitulating human liver-islet axis in normal and disease states	Modeling Type 2 diabetes Drug testing (e.g., metformin)

### Brain organoids-on-chip

**Figure 3.**
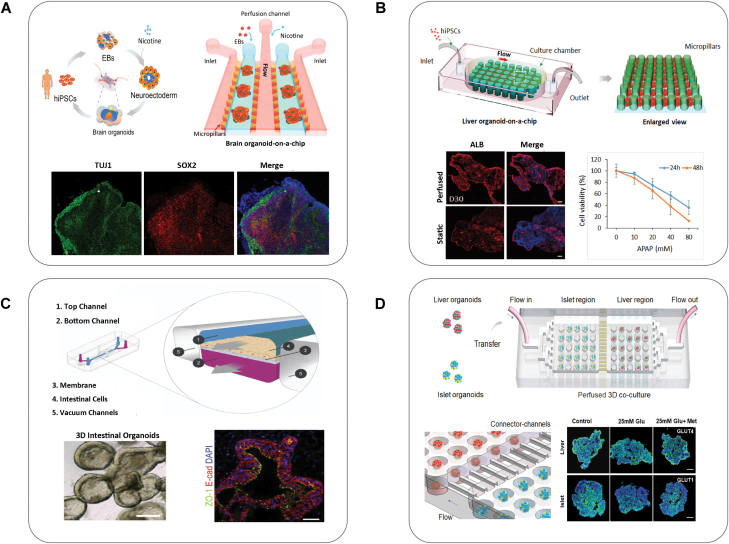
Representative types and functions of organoids-on-chips. (A) A perfusable organ chip was fabricated for *in situ* differentiation and formation of brain organoids from hiPSCs, and this brain organoid-on-a-chip was used to model prenatal nicotine exposure. Reproduced with permission [20]. Copyright 2018, Royal Society of Chemistry. (B) A perfusable liver organoids-on-a-chip derived from hiPSCs were developed for drug testing. Reproduced with permission [44]. Copyright 2018, Royal Society of Chemistry. (C) Human intestinal organoids-derived epithelial cells were incorporated into microengineered chips under continuous media flow, which generated polarized intestinal folds that contained multiple epithelial subtypes and were biologically responsive to exogenous stimuli. Reproduced with permission [42]. Copyright 2018, Elsevier. (D) A perfused multi-organoid system contained two compartmentalized regions enabled the co-culture of liver and islet organoids from hiPSCs, which could recapitulate human-relevant liver-islet axis in normal and Type 2 diabetes states. Reproduced with permission [87]. Copyright 2022, Wiley.

Brain organoids are one of the major progresses in organoids field, holding tremendous potential to study human brain development and neurodevelopmental disorders [88, 89]. However, current brain organoid models displayed high variability in morphology, structure, and maturity, greatly limiting the biological study and downstream applications. Organoid-on-a-chip systems have been developed to engineer brain organoids with well-defined properties. In earlier studies, micropillar array chips were used to form controlled EBs uniform size, facilitating the massive production of brain organoids with reduced variability. Based on this, we designed a perfusable organoid-on-a-chip device with multiply parallel channels that allowed *in situ* differentiation and long-term culture of brain organoids in 3D matrix under fluid flow (Fig. 3A) [20, 45]. We found dynamic cultures could promote neural differentiation and cortical organization in brain organoids-on-chip compared with static cultures in Petri dish. This organ chip system can not only provide efficient nutrients exchange with small volume of culture media, but be amenable to *in situ* imaging, partially addressing the existing limitations. Engineered brain organoids-on-chip also showed great potential to provide new insights into pathogenesis. The prenatal period is highly susceptible to environmental pollutants, possibly leading to genomic dysregulation and neurodevelopmental deficits and diseases with the implications for health of both lifelong and transgenerationally [90]. As such, these engineered brain organoid-on-a-chip systems are promising model to study neurodevelopmental disorders under exposure of various environmental factors, such as nicotine [20], heavy metal cadmium [56], alcohol [77], and valproic acid [91] during embryonic development. It may help us to deeply understand the molecular mechanisms underlying clinical features observed in postnatal neural disorders.

It is worth noting that the spatiotemporal control over the molecular gradients is crucial to direct the organization of diverse brain regions along the axes (e.g., ventral–dorsal axis) as like *in vivo*. The formation of signaling gradient (e.g., WNT, SHH) enabled controlled organization of brain organoids with distinct regions pattern [49, 50]. It might offer a new avenue to guide the dynamic and heterogeneous differentiation of brain organoids by spatiotemporal control the cellular microenvironment. In addition, the absence of supporting cell types or structures (e.g., vascular structure, gliocytes) limits the further maturation of brain organoids. Implementing co-culture of endothelial cells or gliocytes using microfluidic system may support the vascularization of brain organoids and improve their functions. Moreover, a promising set of functional biomaterials including chemical defined hydrogels could be applied to replace the ill-defined Matrigel and further refine the niche of brain organoid development. As such, microfluidic platforms may be essential to facilitate further functional maturation of brain organoids with guided organization, thus advancing their applications in modeling brain development and diseases and translational research.

### Intestinal organoids-on-chip

Intestinal organoids derived from the self-organization of ASC as a pioneering study in organoid field offered a robust model to study intestine physiology, diseases, and pharmacology [92]. Intestinal stem cells located at the basal crypts are characterized by the expression of Lgr5 (leucine-rich G protein-coupled receptor 5), which can generate four major different types of the intestinal epithelium including enterocytes (absorptive), entero-endocrine cells, goblet cells (mucus-secretory), and Paneth cells (lysozyme-producing) via transit-amplifying cells. The Lgr5^+^ intestinal stem cells could give rise to “mini-guts” organoids when cultured with 3D Matrigel and growth factors such as R-Spondin, Noggin, and EGF [23]. The knowledge of intestine development has also led to the establishment of intestine organoids derived from human PSCs, recapitulating many properties of the intestine *in vivo*. However, these intestinal organoids may not fully mimic the morphology and function of native intestine, evidenced by the absence of veritable villous structures that reminiscent of crypt-villus axis. *In vivo*, intestinal peristalsis and contraction were attributed to the epithelial buckling and folding mediated by compressive forces from the smooth muscle cells. As such, intestinal organoids formation in a biomimetic microenvironment may be implicated in proper morphogenesis and physiology of intestine models.

Recently, mechanical cues have been incorporated in engineering intestinal organoids using organ chips technology. For example, an intestine organoid-on-a-chip was developed by co-culture of primary intestinal epithelial cells with endothelial cells under fluid flow and cyclic deformation [41]. By applying cyclic suction to the hollow side chambers, the peristalsis-like motions (10% strain, 0.2 Hz) were generated, which are crucial for maintaining intestinal function. This intestine chip system facilitated the formation of the villi-like intestinal fold structures as well as the differentiation of multi-lineage, recapitulating the key structural features, and functions of native duodenum. In another case, intestinal organoids-derived epithelial cells from hiPSCs were incorporated in a microengineered chip under continuous media flow, which generated polarized intestinal folds with multiple epithelial subtypes (Fig. 3C) [42]. Moreover, the intestine chip is biologically responsive to exogenous stimuli, suggesting the great potential in personal therapy. In future, intestinal organoid-on-a-chip system would be further improved by introducing additional components including immune cells or commensal microbiota [71, 72]. The cross-talk between these factors could contribute to intestinal development and homeostasis, and have great value for host–pathogen research and drug development.

### Liver organoids-on-chip

The PSC-derived liver organoid arises from the differentiation of definitive endoderm lineage and hepatic progenitors, which represent a new type of *in vitro* 3D liver model for disease studies and drug testing. Engineering liver organoids-on-a-chip have offered a promising platform to generate 3D liver tissues with favorable functions in a controlled cellular microenvironment [44, 79]. We have developed a perfusable organ chip with flexible micropillar-based design to generate hiPSC-derived liver organoids in a controlled 3D microenvironment (Fig. 3B) [44]. We found that the fluid flow could promote the differentiation of endoderm and hepatic lineage derived from hiPSCs. Moreover, the generated liver organoids showed improved liver-specific functions, including albumin production and metabolic capabilities under flow. The similar engineered approach was applied for the formation of islet organoids [93] and brain organoids [45, 91] on chip, which enable the whole development process of organoids in a dynamic microenvironment, including controlled EBs formation, *in situ* differentiation and organization of organoids. These systems exhibit the advantages of simplifying tedious manual procedures, reducing variability, and improving functions of organoids.

The established liver organoids-on-chip have been applied for drug testing and liver metabolic disease modeling. In this system, the hepatotoxicity of an oral drug acetaminophen was evaluated [44]. For high-throughput drug screening, this liver organoid system could be improved by further incorporating with additional microfluidic elements for massive organoid formation. Furthermore, this system was used to modeling human nonalcoholic fatty liver disease (NAFLD) by exposure to free fatty acids [80]. The liver organoids displayed a series of typical pathological characteristics associated with steatohepatitis including aberrant triglyceride accumulation, dysfunctional lipid metabolism, and hepatic fibrosis, which may provide insight into the potential mechanisms underlying NAFLD and drug discovery. In the future, the liver organoids-on-chip system may provide a promising platform for the study of host–immune inflammation, virus infectious, and other diseases, as well as individualized medicine.

### Multi organoids-on-chip

In the human body, the process of drug absorption, transport, metabolism, and excretion involves the interactions between multiple organs. To mimic the complex physiological process and systemic responses to external stimuli, it is possible to create multi-organoid system by co-culture of different organoid types in compartmentalized microenvironment using organ chips technology. Recently, we developed a multi-layered organ chip device that enabled the co-culture of hiPSC-derived liver and heart organoids simultaneously [94]. The system was used for the safety assessment of antidepressant drugs, which reflected the hepatic metabolism-dependent cardiotoxicity induced by clomipramine. In addition, multi-organoid system could simulate functional coupling of different organs in physiological-relevant microenvironment, which may be helpful to deeply understand the pathogenesis of systemic diseases at the organ level and advance drug development. Diabetes mellitus (DM) is a complex metabolic disease characterized by chronic hyperglycemia, involving the regulation of glucose homeostasis in the context of connection of multiple organs including liver and pancreatic islets. A liver-islet organoids-on-chip system derived from hiPSCs was recently established by the design of compartmentalized chambers and interconnected microfluidic network, which is beneficial to long-term co-culture of these two organoids and preservation of tissue-specific functions (Fig. 3D) [87]. Upon high glucose condition, this system recapitulated the key pathological features of Type 2 DM and assess the therapeutic effects of hypoglycemic drug metformin. This platform provides a new tool for the study of complex metabolic diseases and systemic responses to drug therapies.

Besides, other types of engineered organoids-on-chip have recently been developed. For example, retinal organoid-on-a-chip was developed to generate complex stratified retinal tissue, recapitulating the interactions between mature photoreceptor segments with retinal pigment epithelium (RPE) [83, 84]. Based on the advances in ocular organoids and tissue chips mimicking different eye tissues (e.g., retina, cornea, and lacrimal gland), organoids-on-chip technology will allow for the study of ophthalmic disease pathogenesis and testing of new therapeutic paradigms in future. Recently, human kidney tubuloids-on-a-chip system was established to enable the formation of polarized kidney tubules with epithelial transport function, which provided a versatile platform study personalized transporter and drug-disposition in tubuloids [86]. By incorporating with additional complex microfluidic elements, future studies will efficiently improve the maturity and functionality of existing organoids, thus advancing their translational applications.

## Conclusions and perspectives

The state-of-the-art organoids-on-chips technology has led to major advancements in understanding organogenesis and diseases, and provided advanced *in vitro* models that could extend the utility of organoids in biomedical applications. This integrative technology has shown the fascinating capacity of growing organoids with higher reproducibility, complicated architecture (e.g., vascular network), and improved function in a controlled manner or in the context of multi-organoid interaction conditions, partially addressing limitations in existing organoid system. Organoids-on-chips can not only offer unique insight into organ physio- and pathological features, but also be conducive to dynamic monitoring of the complex biological processes and high-throughput drug screening by integrating multi-functional analysis methods. This may create exciting possibilities for the development of innovative technology for translational medicine research. With the goal of achieving advanced 3D organ models in higher fidelity, there are still many challenges and opportunities to improve organoids-on-chips for researches in organ developmental biology, personalized therapy, drug screening, and regenerative medicine.

High variability and lack of standardization in methods are both critical issues to be resolved in organoids research. The animal-derived and ill-defined ECM are major limitations in current organoid systems, leading to high variability uncontrolled ECM microenvironment. The xenobiotics may also limit the translational applications of organoids. To address these issues, synthetic matrices or hydrogels that can regulate their biophysical and biochemical properties on demand are being developed. ECM engineering may not only replace poor matrix but also elucidate the ways in which tissues are organized, thus improving the reproducibility of organoids. Several works have designed well-defined hydrogels (e.g., PEG-based hydrogel) for instructing stem cell expansion, differentiation, and organoid formation by dynamically tuning their biochemical components, stiffness, and architecture [95, 96]. Incorporating defined hydrogels into organoids-on-chips might offer new opportunities to produce organoids with higher-order functions and improved reproducibility.

Furthermore, the size of organoids is another critical issue to be concerned. Currently, most organoids can only be obtained at the micrometer to millimeter scale due to the lack of vascularization and limited nutrient exchange at the center of organoids. These organoids are far from human organs at the centimeter scale, possibly leading to low grade of complexity and maturity. This cannot be solved by microfluidic technology alone. The integration of 3D bioprinting with microfluidics is likely to expand the size of chip device in proportion, and scaling up the organoid systems with hierarchical architecture. Moreover, the pre-vascularized scaffolds from anisotropic hydrogels modules may be utilized to enlarge organoid systems with vascular network.

In principle, organoids-on-chips are amendable to live-cell imaging and on-line measurement. Microscopic imaging could be incorporated into this system to facilitate real-time tracking of organoid morphogenesis. Moreover, the integration of multi-omics analysis (e.g., transcriptomics, proteomics) would represent a trend towards deciphering cell fate switches and lineage specification in organoids. This may provide new insights into the research of developmental biology, drug discovery, regenerative medicine, and personalized therapy. Organoids-on-chips can also be integrated with patient-derived stem cells to create personalized disease models and realize precision medicine. Combined with high-throughput analysis, organoids-on-chips would accurately screen and optimize organoid culture conditions, which may provide powerful technical support and guarantee for tissue/organ repair, disease treatment, and advancing the development of regenerative medicine.

**Figure 4.**
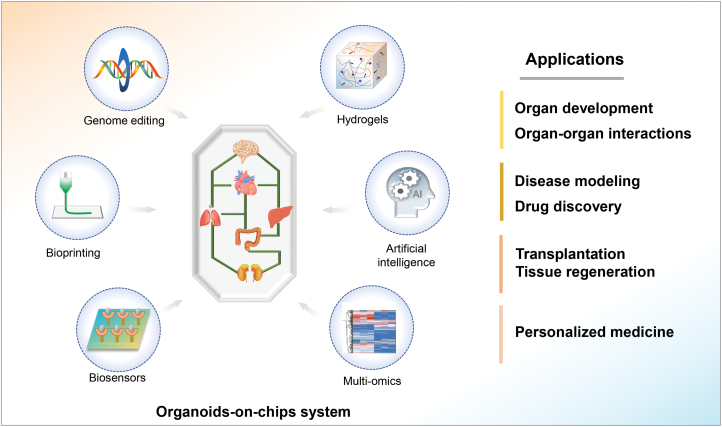
Schematic depiction of advanced organoids-on-chips to meet the needs of biomedical research, extending their applications in organ developmental studies, disease modeling, translational research, and personalized medicine.

At present, organoids-on-chips is a nascent field, which remains much scope for improvement. In the future, organoids-on-chips could be effectively integrated with other cutting-edge technologies (e.g., genome editing, artificial intelligence, etc) to further increase the accuracy with which organoids mirror human physiological processes (Fig. 4). Thus, it also needs cooperative efforts from interdisciplinary researchers and striving for reaching the full utility of organoids in human biomedical studies.
